# Tumor-Derived Microvesicles Modulate Antigen Cross-Processing *via* Reactive Oxygen Species-Mediated Alkalinization of Phagosomal Compartment in Dendritic Cells

**DOI:** 10.3389/fimmu.2017.01179

**Published:** 2017-09-25

**Authors:** Federico Battisti, Chiara Napoletano, Hassan Rahimi Koshkaki, Francesca Belleudi, Ilaria Grazia Zizzari, Ilary Ruscito, Sara Palchetti, Filippo Bellati, Pierluigi Benedetti Panici, Maria Rosaria Torrisi, Giulio Caracciolo, Fabio Altieri, Marianna Nuti, Aurelia Rughetti

**Affiliations:** ^1^Department of Experimental Medicine, Sapienza University of Rome, Rome, Italy; ^2^Department of Molecular and Clinical Medicine, Instituto Pasteur Italia-Fondazione Cenci Bolognetti, Sapienza University of Rome, Rome, Italy; ^3^Department of Gynaecology, Obstetrics and Urology, Sapienza University of Rome, Rome, Italy; ^4^Department of Molecular Medicine, Sapienza University of Rome, Rome, Italy; ^5^Department of Medical and Surgical Sciences and Translational Medicine, Sapienza University of Rome, Rome, Italy; ^6^Azienda Ospedaliera Sant’Andrea, Rome, Italy; ^7^Department of Biochemical Sciences “A. Rossi Fanelli”, Sapienza University of Rome, Rome, Italy

**Keywords:** microvesicles, dendritic cells, antigen-processing, phagosome, radical oxigen species, immune response to cancer, tumor antigens, cancer vaccines

## Abstract

Dendritic cells (DCs) are the only antigen-presenting cells able to prime *naïve* T cells and cross-prime antigen-specific CD8^+^ T cells. Their functionality is a requirement for the induction and maintenance of long-lasting cancer immunity. Albeit intensively investigated, the *in vivo* mechanisms underlying efficient antigen cross-processing and presentation are not fully understood. Several pieces of evidence indicate that antigen transfer to DCs mediated by microvesicles (MVs) enhances antigen immunogenicity. This mechanism is also relevant for cross-presentation of those tumor-associated glycoproteins such as MUC1 that are blocked in HLA class II compartment when internalized by DCs as soluble molecules. Here, we present pieces of evidence that the internalization of tumor-derived MVs modulates antigen-processing machinery of DCs. Employing MVs derived from ovarian cancer ascites fluid and established tumor cell lines, we show that MV uptake modifies DC phagosomal microenvironment, triggering reactive oxygen species (ROS) accumulation and early alkalinization. Indeed, tumor MVs carry radical species and the MV uptake by DCs counteracts the chemically mediated acidification of the phagosomal compartment. Further pieces of evidence suggest that efficacious antigen cross-priming of the MUC1 antigen carried by the tumor MVs results from the early signaling induced by MV internalization and the function of the antigen-processing machinery of DCs. These results strongly support the hypothesis that tumor-derived MVs impact antigen immunogenicity by tuning the antigen-processing machinery of DCs, besides being carrier of tumor antigens. Furthermore, these findings have important implications for the exploitation of MVs as antigenic cell-free immunogen for DC-based therapeutic strategies.

## Introduction

Dendritic cells (DCs) are key regulators of the immune response and are required to induce long-lasting T-cell-mediated cancer immunity (1). Among antigen-presenting cells (APCs), DCs own a unique antigen-presenting machinery that endows them to cross-process and present exogenous antigens, resulting finally in the activation of both CD4^+^ and CD8^+^ T cell response (2).

The biological mechanisms underlying the antigen cross-processing pathways are extensively investigated, also for the full exploitation of DCs for immunotherapeutic intervention (3). Upon internalization by DCs, exogenous antigens are delivered into a complex network of intracellular organelles dynamically interacting among each other. Depending on the type and the physiological characteristics of each organel, antigens undergo to a slow and multistep degradation process that finally allows peptide/HLA class I complex formation. The phagosome of DCs appears to play a fundamental role in exogenous antigen cross-processing by dampening proteases activity, thus reducing protein degradation. In particular, it regulates the export/import of degraded antigens to/from cytoplasm redirecting them to other intracellular pathways for final association to HLA class I and II molecules (4).

*In vivo*, cells release different type of vesicles, distinct for biogenesis, size, lipid composition, and molecular cargo. Microvesicles (MVs) are regarded as conveyors of biological information, able to impact biological behavior of the recipient cells even to distant regions of the body, thus mediating physiological and pathophysiological responses (5, 6). Tumor-derived MVs seem to play a contradictory role in the complex cross talk among cancer cells, immune system, and tumor microenvironment (7, 8). In fact, they can promote tumor growth and foster immunosuppressive mechanisms, resulting in tumor dissemination (9, 10). On the other hand, tumor-derived MVs have been proved to be more immunogenic than the soluble antigens (11) and to induce efficacious anti-tumor immune responses *in vitro* and *in vivo* (12–14), suggesting their possible use as immunogen for DC-based vaccines (15).

We have recently shown that antigen transfer to DCs mediated by tumor MVs is crucially relevant for cross-presentation of tumor-glycosylated antigens such as MUC1. In fact, MUC1 was cross-processed and presented to antigen-specific CD8^+^ T cells only when carried by MVs, while the soluble form of MUC1 was retained in the HLA class II compartment and did not activate any CD8^+^ T cell response (16).

These pieces of evidence prompted us to postulate that tumor-derived MVs could impact the antigen processing in DCs. Using MVs derived from ovarian cancer ascites fluid as well as from a MUC1 engineered tumor cell line, we show that MV internalization modifies DC phagosomal microenvironment, inducing accumulation of reactive oxygen species (ROS) and alkalinization. In addition, we showed that MUC1 cross-processing and presentation to antigen-specific CD8^+^ T cells depend upon these events.

Our results strongly support the hypothesis that tumor-derived MVs convey to DC molecular signals able to reprogram their antigen-processing machinery and not just to transfer antigenic *repertoire*. These findings highlight the potential exploitation of MVs as antigenic cell-free immunogen for DC-based vaccines.

## Materials and Methods

### Recombinant MUC1 Glycoprotein (rMUC1)

The rMUC1 was generated by CHO-K1 cells (ATCC CRL-9618) transfected with a MUC1-murine-IgG_2a_ fusion cDNA construct containing 16 MUC1 tandem repeats. The secreted MUC1-IgG was highly sialylated due to the translational modifications occurring in CHO-K1 cells (17). The rMUC1 glycoprotein was purified from cell culture supernatant by anion exchange chromatography after cleavage of the Fc portion by enterokinase treatment.

### Cell Lines

The lyphoblastoid DG75 cell line (ATCC CRL-2625; kindly provided by Prof. P. Trivedi, “Sapienza” University of Rome) was cultured in RPMI 1640 medium (Sigma-Aldrich, USA, cat n: R0883) supplemented with penicillin 100 U/mL (Sigma Chemical Company, USA), streptomicin 100 µg/mL, 2 mM l-glutamine, 1% non-essential amino acid, 1% sodium pyruvate (all purchased from Sigma), and 10% heat inactivated fetal bovine serum (FBS, Euroclone, Italy, cat n: ECS0180L), 5% CO_2_ at 37°C. MUC1-transfected DG75 lymphoblastoid cells (MUC1-DG75) generated in our laboratory were cultured as above in presence of neomycin (1 mg/mL, Gibco, USA, cat n: 10131-027) (16). Before MV production, MUC1-DG75 cells were analyzed for MUC1 expression by flow cytometry using the MoAb Ma552 clone (Monosan, Netherlands, cat n: MONX10513).

### DCs Generation

Peripheral blood mononuclear cells (PBMCs) of healthy donors were isolated by Ficoll–Hypaque gradient (Lympholite-H, Canada, cat n: CL5020) (Policlinico Umberto I Ethics Committee—Protocol nr. 4214/2016). CD14^+^ monocytes were magnetically immunoselected (StemCell Technologies Inc., CA, USA, cat n: 18058) and cultured in RPMI 1640 (Sigma-Aldrich) + 10% FBS (Euroclone, Italy, cat n: ECS0180L). 50 ng/mL rhGM-CSF and rhIL4 (1,000 U/mL, both from R&D Systems, USA, cat n: 215-GM and 6507-IL, respectively) were added to the cells in culture at days 0 and 2 to obtain immature dendritic cells (iDCs). iDCs were collected at day 5 and used for the experiments. For activation of antigen-specific T cell response assay, iDCs were matured with inflammatory cytokine cocktail: hrTNFα (10 ng/mL; R&D System, cat n: 210-TA), hrIL1β (10 ng/mL; R&D System, cat n: 201-LB), hrIL6 (10 ng/mL; R&D System, cat n: 206-IL), and PGE_2_ 1 µg/mL (Sigma-Aldrich, cat n: P6532).

### MV Purification

Microvesicles were purified from 400 mL of ovarian cancer ascites fluid (MVs_Asc_) (obtained from stages III patients after informed consent from the Department of Gynaecological, Obstetric and Urology—Ethical Committee Protocol nr. 1454/2008) and from the MUC1-DG75 and DG75 cell lines as previously described (16). Briefly, heparinized ascites fluid was centrifuged three times at 4°C and ultracentrifuged twice (10,000 × *g*/30 min; 100,000 × *g*/1 h; 4°C). To produce MVs from the MUC1-DG75 cell line (MVs_MUC1-DG75_) and the untrasfected DG75 cell line (MVs_DG75_), cells were cultured (3.5 × 10^5^ cells/mL) in RPMI 1640 + 2% FBS for 48 h and MVs purified by ultracentrifugation steps starting from 300 mL of secretome for each purification. The culture medium employed for the production of MVs derived from DG75 cell lines (RPMI 1640 + 2% FBS) was processed as before described: the final pellet obtained was used as control of the MVs present in the 2% FBS. Protein concentration was measured by Bradford assay (Bio-Rad Laboratories, USA, cat n: 5000006) with an average of 5.5 µg/µL (MVs_Asc_), 0.9 µg/µL (MVs_MUC1-DG75_), and 1.4 µg/µL (MVs_DG75_).

### Nanoparticles Tracking Analysis (NTA) for MVs

Size determination of MVs_Asc_ and MVs_MUC1-DG75_ was performed by NTA technology (18). MVs were thawed on ice and diluted in PBS between 1:500 and 1:20,000 to achieve the optimal number of MVs/mL. Three videos (30 s each) were recorded for each sample loading, employing the NanoSight NS300 instrument (Malvern Instruments Ltd, Malvern, UK). Measurements were performed employing the NTA 2.3 analytical software. Results were shown as the average of the three recordings.

### Liposomes Preparation

Zwitterionic lipid 1,2-diarachidoyl-sn-glycero-3-phosphocholine (20:0 PC) was purchased from Avanti Polar Lipids (USA cat n: 850368P). Cholesterol (Chol) was purchased from Sigma-Aldrich (cat n: C8667). All lipids were used without further refinement. The liposomal formulation was prepared at desired molar ratio (Chol:PC = 2:8). Lipids were dissolved in chloroform and the solvent was evaporated under vacuum for at least 24 h. Lipid film was hydrated with ultrapure water to obtain a final lipid concentration of 10 mg/mL and then extruded 20 times through a 0.1 µm polycarbonate filter by using the Avanti Mini-Extruder (Avanti Polar Lipids).

### Liposomes Size Characterization

Liposome size was analyzed employing the Zetasizer Nano ZS90 spectrometer (Malvern Instruments Ltd.). To this end, liposomes were diluted 1:100 with distilled water. The same protocol was followed to explore the presence of nano-sized vesicles in the ultracentrifuged culture media.

### Western Blot

MVs_Asc_, MVs_MUC1-DG75_, Liposomes (30 μg/sample), and rMUC1 (1 µg) were separated on 4–12% SDS-PAGE and blotted onto nitrocellulose transfer membrane (Schleicher und Schuell, DE, cat n: ST11306-41BL). Prestained protein ladder by Nippon Genetics Europe GmbH (cat n: MWPO2) was used. Membranes were incubated with anti-MUC1 MoAb Ma552 (Monosan; 1:100, 1 h at room temperature, RT), followed by anti-mouse Fc peroxidase-conjugated antibody [(1:20,000; Jackson ImmunoResearch, USA, cat n: 115-036-062); 1 h at RT]. Protein bands were detected with enhanced chemiluminescence reagents (ECL Western Blotting Detection, Amersham Biosciences, UK, cat n: RPN2106).

### DC Phagosomal pH

Phagosomal pH of monocyte-derived DCs was measured modifying Ref. (19). Briefly, DCs were pulsed in CO_2_-independent medium (Gibco-Life Technologies, UK, #18045-054) with 3 µm microbeads (Polysciences Inc., USA, cat n: 17145-5) coupled with FITC (pH sensitive, Sigma-Aldrich, cat n: F4274) and FluoProbes 647 (pH insensitive, Interchim, France, cat n: FP-BZ8810) (10^6^ cells/100 μL; 30 min, 37°C). After washing to remove beads, cells were incubated at 37°C (“chased”) for the indicated periods of time (10, 20, 30, 60, and 120 min) and immediately analyzed by flow cytometry (FACSCanto II, FACSDiva software, BD Biosciences). A FL1(FITC)/FL4(FluoProbes 647) gate selective for cells that had phagocytosed 1 latex bead was employed. Values were compared with a standard curve obtained by resuspending DCs that had phagocytosed beads in CO_2_-independent medium at a fixed pH (ranging from pH 5.5 to pH 8) containing 0.1% Triton X-100 (Bio-Rad Laboratories, Inc., Italy, cat n: 1610407). Before pulsing with FITC/FP674 coupled microbeads, DCs were pulsed (30 min, 37°C) with: rMUC1 glycoprotein (20 µg/mL), MVs purified from ovarian ascites fluid or from MUC1-DG75 cells line (500 µg/mL), or with Liposomes (2.5 mg/mL). To block NADPH oxidase 2 (NOX2) activity, 10 µM Diphenyleneiodonium chloride (DPI, Sigma-Aldrich, cat n: D2926) was added to DCs 30 min before MVs pulsing and it was maintained throughout the experiment.

### ROS Detection in DC Phagosome and in MVs

Phagosomal ROS of DCs following MV uptake was measured modifying the protocol by Savina et al. (19). Cells were pulsed with 3 µm microbeads (Polysciences Inc.) coupled with Dihydrorhodamine 123 (DHR123; ROS sensitive, Life Technologies, cat n: D-632) and FluoProbes 647 (ROS insensitive, Interchim) (10^6^ cells/100 μL, 15 min, 37°C), in CO_2_-independent medium (Gibco-Life Technologies). After pulsing with DHR123/FP647 coupled microbeads, DCs were pulsed with MVs purified from ascites fluid or from MUC1-DG75 cells line (500 µg/mL), with rMUC1 glycoprotein (20 µg/mL) or with Liposomes (2.5 mg/mL) (15 min, 37°C). After washing, cells were incubated at 37°C (“chased”) for the indicated periods of time (0, 10, 20, 30, and 60 min) and immediately analyzed by flow cytometry (FACSCanto II, BD Biosciences), using a FL1 (DHR123)/FL4(FluoProbes 647) gate selective for cells that had phagocytosed 1 latex bead. To enhance or block phagosomal ROS production, phorbol 12-myristate 13-acetate (PMA, 0.5 µg/mL, Sigma-Aldrich, cat n: P8139) and DPI (5 µM, Sigma-Aldrich), respectively, were added to DCs 30 min before pulsing and were kept throughout the experiment. ROS values were obtained by comparing the DHR123/FP647 ratio of pulsed and unpulsed DCs ± DPI/PMA at different time of chase (10, 20, 30, and 60 min) with the unpulsed DCs at time 0 of chase.

Radical content in MVs was determined by 2′,7′-dichlorofluorescin diacetate (DCF-DA, Sigma-Aldrich, cat n: D6883) staining. MVs from ascites fluid and MUC1-DG75 were incubated in PBS + 10 mM DCF-DA (40 min, 37°C in the dark). After washing with PBS, samples were analyzed by flow cytometry (FACSCanto II, BD Biosciences). Fluorescent Nile Red particles (Spherotech, US, cat n: FP-0256-2) with size of 100–300 nm were used as size marker. Protein-empty Liposomes were used as negative control. To test the overall presence of ROS generating molecules 10 mM H_2_O_2_ was added (Sigma-Aldrich, cat n: H1009).

### Immunofluorescence Microscopy

After pulsing with MVs_Asc_, DCs ± DPI were cytospun (8 × 10^4^ cells/sample) and fixed with cold acetone/methanol (1:1; VWR Chemicals, France, cat n: 2006-321 and 200659-6, respectively). After blocking with 20% Bovine Serum Albumine (Sigma-Aldrich, cat n: A2153), DCs were incubated (45 min) with the anti-MUC1 MoAb Ma552 (1:20) followed by Alexa 488-conjugated goat anti-mouse F(ab′)_2_ (1:100, 30 min; Jackson ImmunoResearch, cat n: 115-546-008). After blocking and washing, the samples were incubated with PE/Texas red-conjugated HLA-II-DR (30 min in the dark, Beckman Coulter, cat n: IM3636) to detect the HLA class II compartment. To visualize HLA class I compartment, the following reagents were employed: the rabbit polyclonal antibody anti-calreticulin (1:50, 45 min; Stressgen, US, cat n: SPA-600) followed by Texas red-conjugated goat anti-rabbit antibody (1:200, 30 min in the dark; Jackson ImmunoResearch, cat n: 111-075-144) and anti-HLAI MoAb W6/32 (culture supernatant) followed by Cyanine Cy™3 affiniPure F(ab′)_2_ fragment goat anti-mouse IgG (Jackson ImmunoResearch, cat n: 715-166-150, 1:1500). Calreticulin/EEA1 intracellular distribution was evaluated employing: anti-calreticulin polyclonal antibody and anti-EEA1 MoAb (BD Transduction Laboratories, cat n. 610456, 1:50) revealed by FITC-conjugated goat anti-rabbit IgG (Jackson ImmunoResearch, cat n: 111-096-144, 1:200) and Cyanine Cy™3 affiniPure F(ab′)_2_ fragment goat anti-mouse IgG (Jackson Immuno Research), respectively. Unpulsed DCs were used as negative control. All incubations were carried out at RT. Fluorescence signals were visualized with an Axiovert 200 inverted microscope (Zeiss, Germany); cells were scanned in a series of 0.5 µm sequential sections with an ApoTome System (Zeiss) and images were all acquired by the digital camera Axio CAM MRm (Zeiss). Image analysis was performed by the Axiovision software (Zeiss) and reconstruction of a selection of three central optical sections was shown in each figure. Quantitative analysis of the extent of colocalization of fluorescence signals was performed using the KS300 3.0 Image Processing System (Zeiss). The mean ± SD percent of colocalization was calculated analyzing a minimum of 30 cells for each treatment randomly taken from three independent experiments.

### MUC1^+^ CD8^+^ T Cell Enrichment and IFNγ-ELISpot

MUC1-enriched CD8^+^ T cells were expanded in culture from PBMCs of a MUC1 vaccinated ovarian cancer patient (open-label phase I/II safety clinical peptide vaccination trial, approved by Policlinico Umberto I Ethics Committee and Italian National Institute of Health/protocol no. LITRM/DIMIGE05/01; Ethical Committee Protocol nr. 1454/2008) (20). Briefly, PBMCs were isolated by Ficoll/Hypaque density gradient and CD8^+^ cells were immunoselected (Stemcell Technologies, USA, cat n: 18053) and kept in RPMI + 5% FCS. CD8^−^ cells (2 × 10^6^ cell/mL) were pulsed overnight (o/n) with 50 µg/mL of MUC1_159-167_ peptide (SAPDNRPAL) (ClinAlfa, Switzerland, C-S-270/ACO598) and 5 µg/mL β2-microglobulin (Sigma Aldrich, cat n: 4890) in RPMI, 1% FCS. CD8^−^ cells were then irradiated (30 Gy) and plated with autologous CD8^+^ T cells (1:1; 2 × 10^6^ total cells/mL) in RPMI + 5% FCS, supplemented with IL2 (50 UI/mL; Prepotech, USA, cat n: 200-02) and IL7 (10 ng/mL; R&D System, cat n: 207-IL). After 7 days, freshly isolated and MUC1-pulsed autologous PBMCs were irradiated and added to the culture (1:1). At the same time, autologous CD14^+^ cells were isolated and differentiated into iDCs as above described. At day 5, iDCs were harvested: half of the iDCs were incubated with 10 nM DPI for 30 min in RPMI 1640 for DC culture (2 × 10^6^/mL). iDCs were then pulsed o/n with MVs from the DG75 cells (400 µg/mL), MVs from MUC1-DG75 cells (400 µg/mL) and MUC1_159-167_ peptide with β2-microglobulin (50 and 5 µg/mL, respectively), in the presence or absence of 10 nM DPI. After 4 h, iDCS were matured with the cytokine cocktail o/n. mDCs were then washed and added to MUC1^+^ enriched CD8^+^ T cells (1:5), previously expanded in culture and purified by Ficoll/Hypaque density gradient to remove cell debris. DCs/T cells were plated (2 × 10^6^cell/mL) in duplicate o/n in the anti-IFNγ-precoated (1:200; BD Biosciences, cat n: 51-2555KZ) ELISpot plate (MultiScreen, Merck, Germany, cat n: S2EM004M99). Unpulsed DCs + T cells samples were used as negative control. IFNγ cytokine release was detected with biotinylated anti-IFNγ antibody (1:250, 2 h; BD Bioesciences, cat n: 51-1890KZ), revealed with streptavidin-alkaline phosphatase (BD Biosciences 554065) (1:1,000, 100 mL/well, 1 h) and chromogen substrate (Sigma, B5655). Spots were counted using the ImmunoSpot Image Analyzer (Aelvis, Germany). Average value of background control samples (unpulsed DCs + T cells) was subtracted from average value of each experimental condition. IFNγ response was considered positive adopting as cutoff a twofold increase (IFNγ production induced by DCs + MVs_MUC1-DG75_/IFNγ production induced by DCs + MVs_DG75_).

### ELISA

Flat-bottomed 96-well EIA/RIA plates (Costar, USA, cat n: 3690) were coated with 2 µg/well of MV_Asc_ and MVs_MUC1-DG75_ in Carbonate Buffer o/n. Plates were washed and incubated with PBS-5%BSA (Sigma-Aldrich, cat n: A4503). After three washes in PBS-1%BSA-0.01% Tween 20 (Biorad, cat n:1706531), samples were incubated for 1 h, 37°C with relevant antibody, in duplicates. The following MoAbs were used at 2 µg/mL, when otherwise specified: anti-CD9 (Abcam, England, ab58989), anti-GM130 (SantaCruz Biotechnologies, USA, cat n: sc-55591), MOPC21 (Sigma-Aldrich, M-7894), MoAb Ma552 clone (Monosan, 1:100).

The polyclonal rabbit Abs used were: anti-CD63 (SantaCruz Biotechnologies, cat n: sc-15363, 1:200), anti-TGN46 (Sigma, USA, cat n: T7576, 1:1,500); Normal Rabbit Serum (NRS) (ThermoFisher Scientific, USA, cat n: 10510; 1:1,000). Peroxidase-conjugated affinity purified F(ab′)_2_ goat anti-mouse IgG (H + L; 1:5,000, Jackson ImmunoResearch cat n: 115-036-062) or affinity purified F(ab′)_2_ goat anti-rabbit (H + L; 1:5,000, Jackson ImmunoResearch cat n: 111-036-045) was added to wells (1 h RT), washed and followed by chromogen addiction (5 mg *O*-phenylenediamine, Sigma, cat n: P6912) in citrate phosphate buffer 10 mL and 30% H_2_O_2_ 10 µL (Sigma-Aldrich). The absorbance was measured at 492 nm. Sample ODs were subtracted of the OD values of control samples (MOPC1 and NRS OD value for MoAb and rabbit antibodies, respectively).

### Statistical Analysis

Statistics was performed using GraphPad Prism software, version 6 (GraphPad Software, Inc., USA). Results were expressed as mean values ± SD. *p* values were calculated using Student’s *t-*test when comparing two groups of continuous variables. Significance level was defined as *p*-value <0.05 (*p < 0.05; **p < 0.01; ***p < 0.005).

## Results

### MV Uptake Induces Alkalinization in DC Phagosomal Compartment

In DCs, alkalinization of the phagosomal compartment seems to be crucial for efficient antigen cross-processing and presentation (4). We have previously shown that the tumor-associated MUC1 is cross-processed by DCs when the glycoantigen is carried by MVs, not when MUC1 tumor-associated glycoprotein is internalized as soluble molecule (16). We hypothesized that MVs could impact phagosome microenvironment, thus signaling to DCs for a distinct antigen-processing pathway.

**Figure 1:**
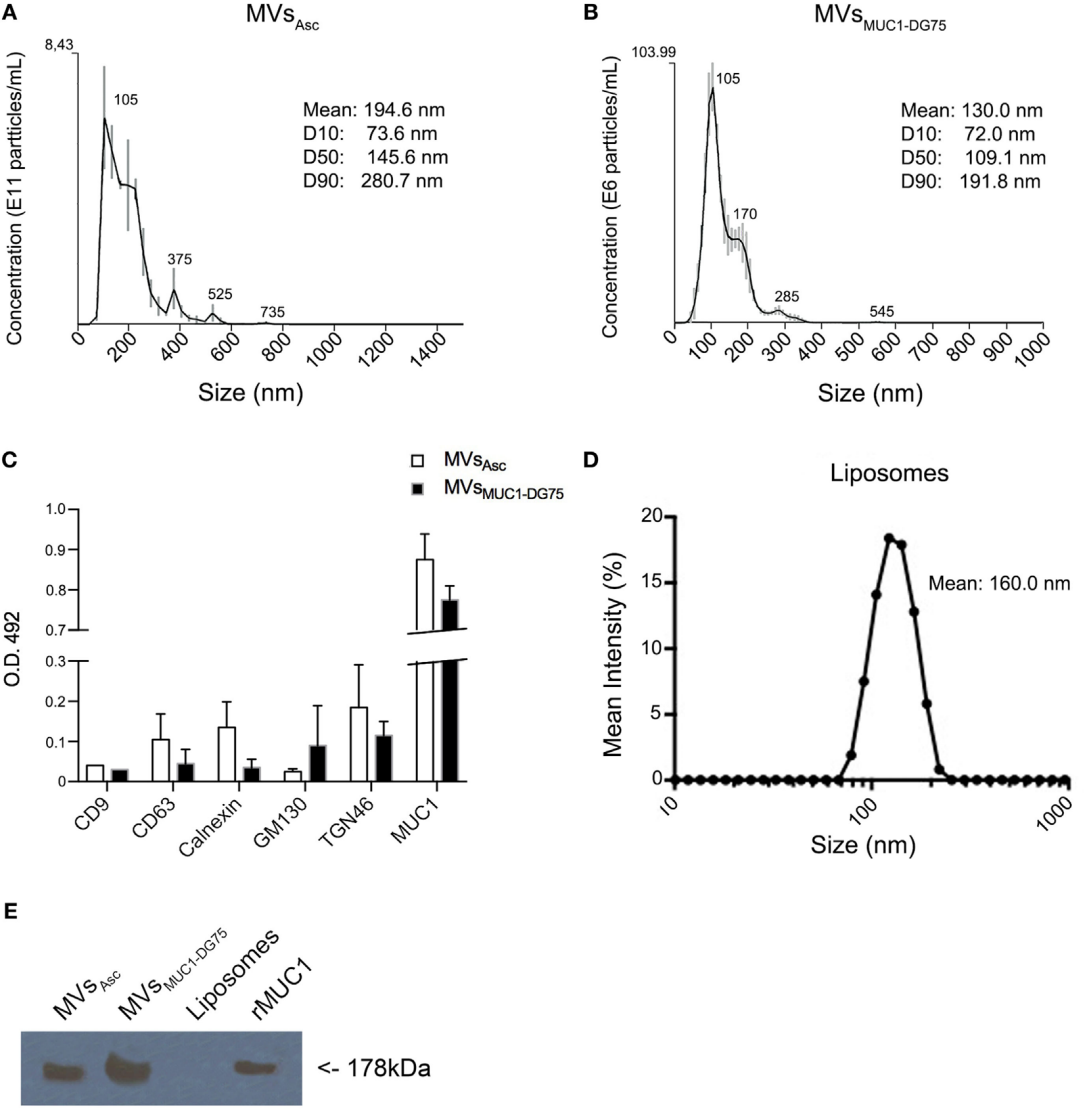
Characterization of tumor-derived microvesicles (MVs) and Liposomes. **(A,B)** Size measurement of MVs derived from ovarian cancer ascites fluid [MVs_Asc_; **(A)**] and shed by the MUC1-transfected DG75 lymphoblastoid cell line [MVs_MUC1-DG75_; **(B)**] using Nanosight NS300 that employs nanoparticles tracking analysis (NTA) technology. Results are plotted as graph; *y*-axis: concentration of particles; *x*-axis: size of particles in nanometer. The black curve is obtained by the merge of three independent measurements for each MV sample. SD and the medium sizes of identified vesicle subsets are shown above the merged curve. Legend shows the particle medium size of the entire sample and the dimension of the 10, 50, and 90% of vesicles in the sample (D10, D50, and D90, respectively). **(C)** CD9, CD63, Calnexin, GM130, TGN46, and MUC1 proteins expression in MVs_Asc_ (white histograms) and MVs_MUC1-DG75_ (black histograms) as detected by ELISA assay (O.D. 492). Sample ODs were subtracted of the OD values of control samples [MOPC1 and Normal Rabbit Serum (NRS)]. Results are plotted as average and SD of two independent experiments. **(D)** Liposomes size measurement using Zetasizer Nano ZS90 that employs NTA technology. Results are plotted as graph; *y*-axis: mean intensity percentage; *x*-axis: size of particles. The black curve shows the representative Liposomes dimension out of five different size measurements. **(E)** MUC1 expression in vesicle samples. MUC1 was detected by Western Blot analysis employing the MoAb Ma552. MVs_Asc_ and MVs_MUC1-DG75_ were positive, while Liposomes were negative. The recombinant MUC1 glycoprotein (rMUC1) was used as positive control.

To test this hypothesis, distinct tumor MV formulations were employed. Cell-derived extracellular vesicles were purified by ultracentrifugation from ovarian cancer ascites fluid and from the supernatant of the MUC1-DG75 cell line, a lymphoblastoid cell line engineered to express MUC1 also in the MVs (16). Size analysis of MV samples was performed by NTA technology. Ovarian cancer ascites fluid-derived vesicles (MVs_Asc_) were mostly distributed within the range between 105 and 375 nm, with an overall average size of 194.6 nm (Figure 1A). Extracellular vesicles from MUC1-DG75 cell line (MVs_MUC1-DG75_) were also heterogeneous for size, as previously characterized by transmission electron microscopy (TEM) (16, 21). NTA analysis showed two main vesicles subsets having a medium size of 105 and 170 nm, respectively (mean size: 130 nm) (Figure 1B).

The MVs were characterized for the presence of intracellular compartment markers. Exosome markers such as CD9 and CD63 as well as calnexin (ER marker) and GM130 (Golgi marker) were expressed at low levels (Figure 1C) in both vesicle samples. TGN46, marker for trans-Golgi network compartment, was also detected. The MUC1 tumor antigen was present at high levels in both vesicle samples as detected by ELISA and WB (Figures 1C,E).

These results suggest that extracellular vesicles obtained were heterogenous for size and intracellular origin, with a prevalence of vesicles derived from the plasma membrane/exocytic pathway as indicated by the size (>100 nm) and the presence of MUC1 and TGN46 markers.

As “protein free vesicles,” Liposomes were produced with a medium size of 160 nm, corresponding to the average size of the tumor-derived MVs (Figure 1D). Liposomes were made by Zwitterionic lipid 1,2-diarachidoyl-sn-glycero-3-phosphocholine andChol to resemble the lipid pattern of plasma membrane. Also the rMUC1 carrying tumor-associated glycans (22) was employed as soluble MUC1 form (Figure 1E).

**Figure 2:**
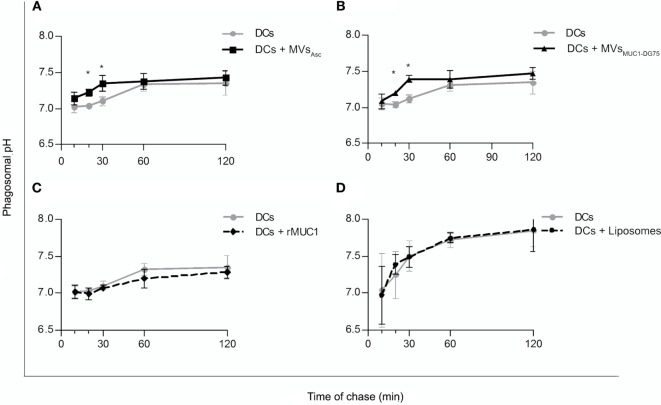
Increase of dendritic cell (DC) phagosomal pH following internalization of tumor-derived MVs. Time course experiments to evaluate the phagosomal pH values in unpulsed DCs (gray line) and DCs pulsed with MVs_Asc_ **(A)**, MVs_MUC1-DG75_ **(B)**, recombinant MUC1 glycoprotein (rMUC1) **(C)**, and Liposomes **(D)** (black line). Each graph shows the average and SD of independent experiments. DC phagosomal pH significantly increased (*p < 0.05) when DCs were pulsed with MVs purified from ovarian cancer ascites fluid [**(A)**, five independent experiments] or shed by DG75 cell line [**(B)**, three independent experiments] (*p < 0.05 at 20 and 30 min). No change in phagosomal pH was observed when rMUC1 glycoprotein [**(C)**, 5 independent experiments] or Liposomes [**(D)**, four independent experiments] were used to pulse DCs.

Dendritic cells were pulsed with MVs_Asc_, MVs_MUC1-DG75_, Liposomes, and rMUC1, and phagosomal pH was monitored for 2 h by flow cytometry (Figure 2). Unpulsed DCs, employed as control, showed a neutral phagosomal pH (7.01–7.03 pH) for the first 20 min of chase that increased up to 7.32 ± 0.081 SD pH at 60 min. When DCs were pulsed with MVs_Asc_ (DCs + MVs_Asc_), phagosomal pH significantly increased compared to unpulsed DCs already at 20 min, reaching 7.34 ± 0.108 SD pH (*p* < 0.05) after 30 min of chase. After 1 h, pH reached similar values in both DCs and DCs + MVs_Asc_ (Figure 2A). Similar pH kinetic was observed when DCs were pulsed with MVs_MUC1-DG75_: again a significant increase of phagosomal pH was observed within the first 30 min of chase (7.39 ± 0.05 SD pH, *p* < 0.05) and similar pH values for both samples were reached after 1 h of chase (Figure 2B). To exclude that this effect was due to vesicle contaminant in the bovine serum, DCs were incubated with the pellet fraction obtained by the ultracentrifugation of the culture medium (RPMI + 2% FBS) and displayed a pH kinetic similar to the untreated DCs (Figure S1A in Supplementary Material). Results of size analysis of this pellet were not compatible with the presence of vesicles, but they corresponded to a typical size distribution of proteins enriching culture media (23) (Figure S1B in Supplementary Material).

Uptake of soluble rMUC1 glycoprotein and the protein free Liposomes did not affect phagosomal pH (Figures 2C,D, respectively), suggesting that the rapid DC phagosomal alkalinization observed is a specific response to uptake of the tumor-derived MVs.

### MV Internalization by DCs Induces Increase of ROS Levels in DC Phagosomal Compartment

**Figure 3:**
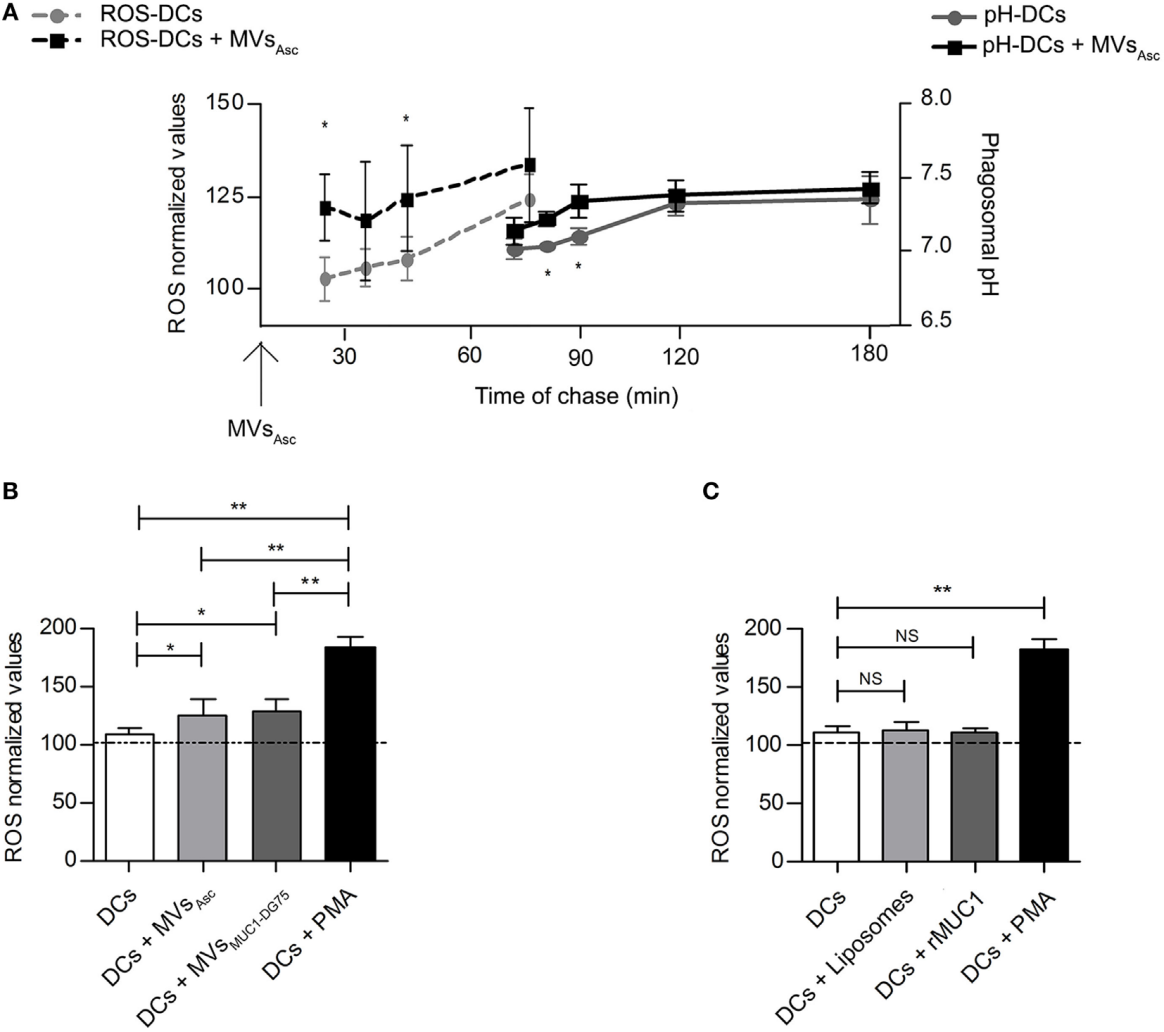
Phagosomal reactive oxygen species (ROS) molecules increase following dendritic cell (DC) uptake of tumor microvesicles (MVs) shortly before phagosomal alkalinization. **(A)** Modulation of the phagosomal ROS (dotted lines) and pH (continuous lines) in DCs following internalization of MVs_Asc_ considered as T0 for both the kinetic measurements. Results are plotted as average of 4 independent experiments for ROS analysis and 5 independent experiments for phagosomal pH analysis. SD and statistical significance are also shown (*p < 0.05). **(B)** ROS measurement at 30 min of chase; MV pulsed DCs (light and dark gray histograms for DCs + MVs_Asc_ and DCs + MVs_DG75-MUC1_ samples, respectively) had a significant higher ROS content compared to unpulsed DCs (white histogram, *p < 0.05). PMA-treated DCs (black histogram) are used as positive control. ROS levels are plotted as arbitrary units of three independent experiments (three donors) normalized toward the unpulsed DCs at time 0 of chase (100 arbitrary units). **(C)** ROS measurement at 30 min of chase; DCs pulsed with Liposomes (light gray histogram) and with the soluble recombinant MUC1 glycoprotein (rMUC1) glycoprotein (dark gray histogram) did not show any difference in term of ROS content compared to unpulsed DCs (white histogram). PMA-treated DCs (black histogram) were used as positive control. ROS levels are plotted as arbitrary units of three independent experiments (three donors). ROS level in unpulsed DCs at time 0 of chase correspond to 100 arbitrary units (dotted line). *p < 0.05; **p < 0.01.

Since phagosomal alkalinization seems to be strictly dependent on reactive oxygen species (ROS) generation and consequently protons consuming (24), the level of ROS into the DC phagosomal compartment was measured following MVs_Asc_ internalization in time course experiments (Figure 3A, dotted lines). Phagosomal radical species were consistently higher in MVs_Asc_ pulsed DCs than in unpulsed DCs, being statistically significant in the first 30 min of chase.

It appeared that ROS increase occurred shortly before the pH alkalinization (Figure 3A, continuous lines), if considering the MVs_Asc_ pulsing of DCs as T_0_ for both phagosomal ROS and pH measurement. Similar results were obtained when DCs were pulsed with MVs_MUC1-DG75_ (data not shown). This might suggest that MVs of tumor origin trigger phagosomal ROS production and pH increase as consequence.

Reactive oxygen species modulation in DCs was then analyzed at 30 min of chase after pulsing with tumor-derived MVs (MVs_Asc_ and MVs_MUC1-DG75_; Figure 3B) and with both Liposomes and the soluble rMUC1 (Figure 3C).

When both MVs_Asc_ and MVs_MUC1-DG75_ were used to pulse DCs, ROS significantly increased (*p* < 0.05) as compared to unpulsed DCs, although at a lower extent than DCs treated with PMA, used as stimulus to maximize ROS production (*p* < 0.01) (Figure 3B). No change in radical species was observed when Liposomes or soluble rMUC1 glycoprotein were employed (Figure 3C). These results indicate that only tumor-derived MVs could modulate ROS accumulation into the phagosomal compartment of DCs, probably inducing the DC phagosomal alkalinization.

### Tumor MVs Carry ROS Species and Contribute to Phagosomal Alkalinization

These results prompted us to investigate whether tumor-derived MVs carried endogenous source of radical species. Liposomes, MVs_Asc_ and MVs_MUC1-DG75_ were first identified by SSC-FSC analysis in flow cytometry and gated employing 100–300 nm beads as reference (Figure S2 in Supplementary Material). These measurements were in agreement with the MV size distribution as determined by NTA analysis (Figure 1).

**Figure 4:**
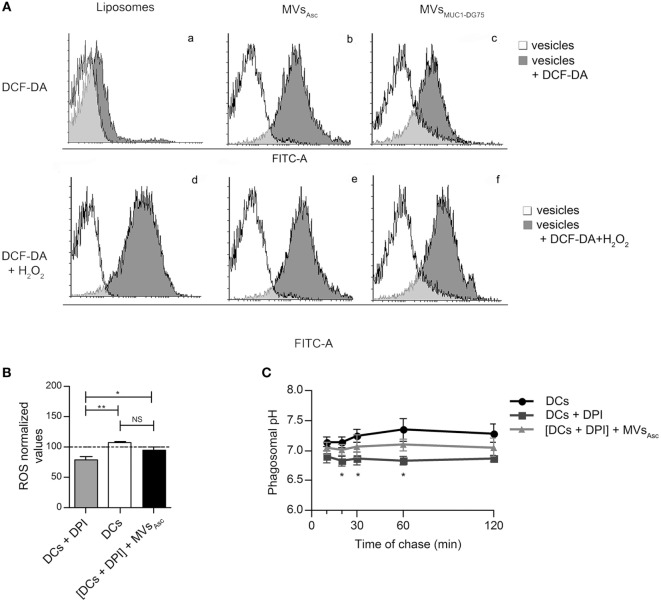
Tumor microvesicles (MVs) are source of radical species and modulate phagosomal alkalinization. **(A)** The presence of reactive oxygen species (ROS) molecules in Liposomes (a, d), MVs_Asc_ (b, e), and MVs_MUC1-DG75_ (c, f) was analyzed used DCF-DA (a–c) and DCF-DA + H_2_O_2_ as positive control (d–f). Results are plotted as graph; *y*-axis: event count; *x*-axis: Log-FITC-A (from 0 to 10^5^). White histograms are referred to the background fluorescence of Liposomes and MVs samples, gray histograms represent Liposomes and MVs treated with DCF-DA (a–c) or with DCF-DA + H_2_O_2_ (d–f). The mean fluorescence intensity for untreated sample, DCF-DA-treated and DCF-DA + H_2_O_2_ samples were, respectively: MFI 2, 18, 229 for Liposomes; MFI 5, 381, 481 for MVs_Asc_; MFI 9, 75, 212 for MVs_MUC1-DG75_. **(B)** ROS species measurement in DPI-treated DCs following MVs_Asc_ pulsing. MVs_Asc_ uptake (black histogram) restored ROS levels in DPI-treated DCs (gray histogram) similarly to unpulsed DCs (withe histogram). ROS levels are plotted as arbitrary units of three independent experiments (three donors). ROS level in unpulsed DCs at time 0 of chase correspond to 100 arbitrary units. *p < 0.05; **p < 0.01. **(C)** Phagosomal pH measurement in DCs in the presence of DPI, without or with MVs_Asc_ pulsing. Diphenyleneiodonium chloride (DPI) treatment decreased pH compared to untreated DCs (black line, squared vs. black line, dotted). In MVs pulsed DPI-treated DCs (gray line, triangle) phagosomal pH was partially restored. The difference between DPI-treated DCs (black line, squared) and MVs pulsed DPI-treated DCs (gray line, triangle) is statistically significant from 20 to 60 min of chase (*p < 0.05). Values are mean ± SD of three independent experiments.

The radical species content of Liposomes, MVs_Asc_ and MVs_MUC1-DG75_ was measured by staining with the ROS sensitive dye DCF-DA. Results indicated that while Liposomes remained negative, MVs_Asc_ and MVs_MUC1-DG75_ were positive for DCF-DA staining while (Figure 4A, a–c, respectively). The exogenous addition of H_2_O_2_ induced appearance of staining in Liposomes sample (13.02-fold MFI increase, Figure 4A, d) and a slight increase of reactivity in MVs_Asc_ and MVs_MUC1-DG75_ (1.25- and 2.82-fold MFI increase, respectively, Figure 4A,e,f, respectively).

To assess whether radical species carried by tumor MVs functionally impacted DC phagosomal activity, DCs were treated with DPI, specific inhibitor of NOX2, a key enzyme in the fine tuning of ROS production in DCs (25). As expected, ROS level significantly decreased (*p* < 0.01) in DPI-treated DCs as compared to untreated DCs (Figure 4B). Addition of MVs_Asc_ to DPI-treated DCs significantly restored radical species (*p* < 0.05) at similar levels to untreated DCs (Figure 4B). When pH was measured in time chase experiments, DPI treatment induced phagosomal acidification (*p* < 0.01) throughout the experiment (10–120 min) (Figure 4C). Pulsing of DPI-treated DCs with MVs_Asc_ significantly increased the pH values in the first 60 min (*p* < 0.05), restoring alkalinization in the phagosome at pH values close to untreated DCs (Figure 4C). Similar results were obtained when DCs were pulsed with MVs_MUC1-DG75_, while no change was observed when Liposomes were employed (data not shown). These results indicate that the phagosomal alkalinization upon MVs’ internalization is dependent upon ROS increase.

### DC Phagosomal Alkalinization Induced by Tumor MVs Enhances MUC1 Processing to HLAI Compartment and Cross-Presentation of MUC1 Antigen Requires Functional DCs

In order to clarify the physiological role of phagosomal alkalinization induced by tumor MVs in MUC1 antigen cross-processing, the intracellular re-localization of MUC1 was analyzed by immunofluorescence in DCs pulsed with MVs_Asc_, treated or not with DPI.

After 12 h pulsing, MUC1 detection was strongly reduced in DCs treated with DPI (*p* < 0.005) as compared to untreated DCs, suggesting that the forced acidification induced by DPI had impacted degradation of the MUC1 (Figure S3A in Supplementary Material).

**Figure 5:**
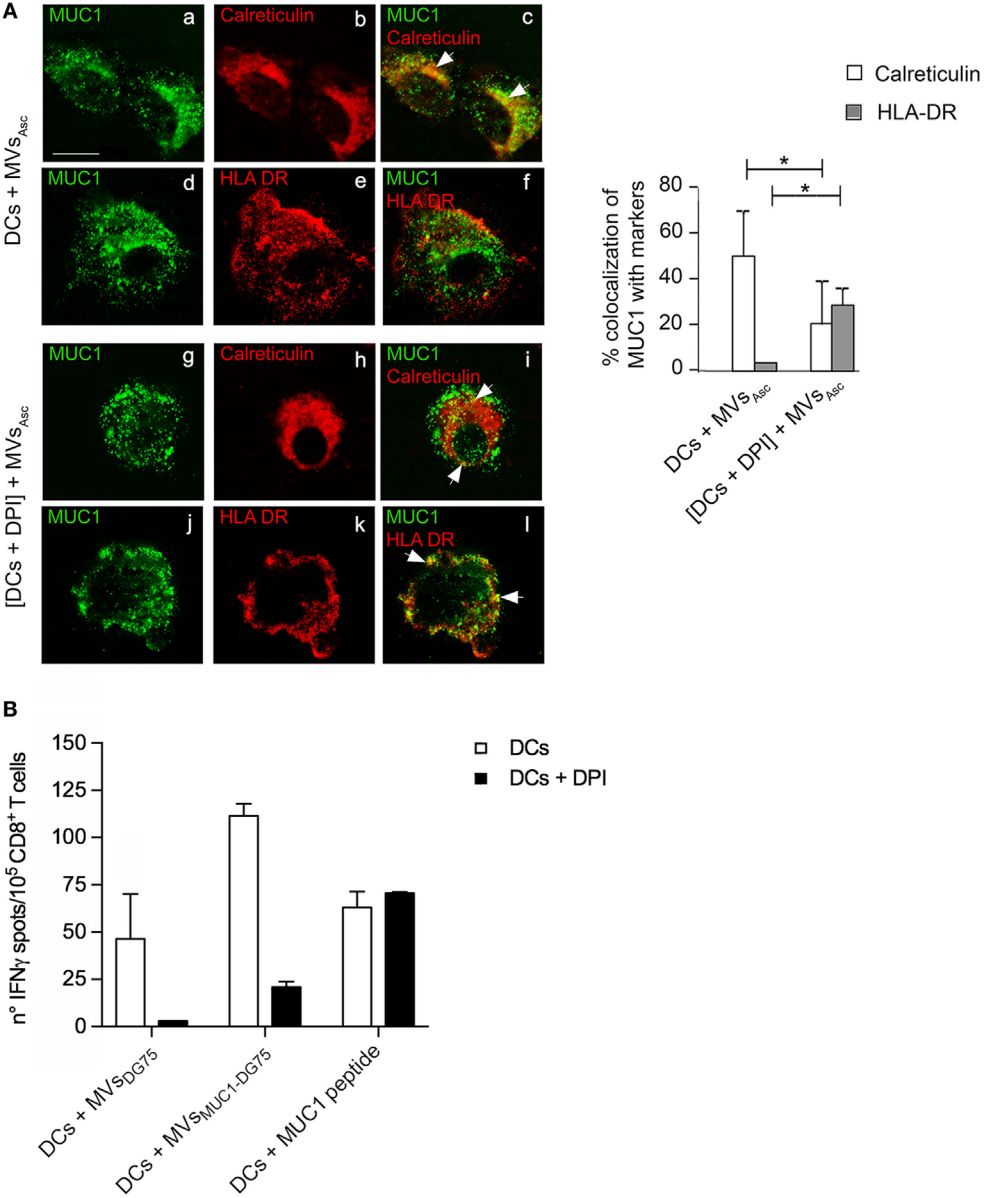
Tumor microvesicles (MVs) induce MUC1 processing to HLAI in dendritic cells (DCs) with acid phagosomal compartment, while cross-presentation of MUC1 antigen requires functional DCs. **(A)** Intracellular localization of MUC1 carried by MVs_Asc_ in DCs (a–f) and in diphenyleneiodonium chloride (DPI)-treated DCs (g–l) was visualized by immunofluorescence staining after 12 h of internalization employing the anti-MUC1 MoAb Ma552 (green, a, d, g, j) and antibodies direct against specific markers for intracellular compartments (red). In particular: anti-calreticulin rabbit polyclonal antibody for ER (b, c, h, i) and anti-HLAII-DR for HLA-II compartment (e, f, k, l). Colocalization of the green and red immunofluorescence signals is shown in yellow (c, f, i, l). Quantitative analysis of colocalization was calculated as reported in material and methods and plotted as histograms. Results are expressed as mean values ± SD. Student’s *t-*test was performed and significance level has been defined as *p* < 0.05. *p < 0.05. Bar: 10 µm. **(B)** ELISpot assay to evaluate the IFNγ cytokine secretion by MUC1-specific enriched CD8^+^ T cells from an ovarian cancer patient in response to mDCs (white histograms) and mDCs + DPI (black histograms) pulsed with MVs_DG75_, MVs_MUC1-DG75_ and MUC1_(159-167)_ peptide. Results are shown as average and SD of replicates.

In DPI-untreated DCs, MUC1 mostly colocalized with calreticulin (53%), ER protein employed as HLA class I pathway marker (Figure 5A, a–c). The MUC1 antigen was scarcely associated with HLA-II compartment marker HLA-II-DR (<10%) (Figure 5A, d–f).

Diphenyleneiodonium chloride treatment also affected MUC1 intracellular distribution: MUC1/HLAII-DR colocalization significantly increased (38%, *p* < 0.05) and was distributed in intracellular dots strongly suggesting that HLA class II pathway was engaged (Figure 5A, j–l). Conversely, MUC1 distribution was reduced, although not abolished, in calreticulin compartment (25%, *p* < 0.05) (Figure 5A, g–i). Similar results were obtained when the HLA class I molecule was employed as compartment marker (Figure S3B in Supplementary Material). No overlap between calreticulin^+^ compartment and early endosome (EEA1^+^) compartment was observed (Figure S3C in Supplementary Material). When DCs (±DPI treatment) were pulsed with MVs_MUC1-DG75_, similar MUC1 intracellular distributions in HLA class I and class II compartments were observed as compared to MVs_Asc_ pulsed DC cells (data not shown).

The impact of reduced MUC1 antigen cross-processing on antigen presentation was analyzed as MUC1-specific T cell responses.

MUC1-specific CD8^+^ T cells were expanded from PBMCs of an ovarian cancer patient, previously vaccinated with the HLAI-A2-restricted MUC1_159-167_ peptide (20). Autologous DCs (±DPI treatment) were pulsed with MVs_MUC1-DG75_, MVs_DG75_ (MVs from untransfected DG75 cells) or loaded with the immunogenic MUC1_159-167_ peptide. Unpulsed and pulsed DCs were used as APCs to stimulate the enriched MUC1_159-167_ specific CD8^+^ T cells. T cell activation was evaluated as IFNγ release in ELISpot assay. As Figure 5B reports, DCs + MVs_MUC1-DG75_ induced a stronger IFNγ T-cell mediated response than the one induced by DCs + MVs_DG75_, indicating that specific antigen response occurred, in agreement with what previously reported (16).

When DCs were pulsed in the presence of DPI, the IFNγ response to DCs + MVs_MUC1-DG75_ drastically dropped (80% reduction) and was abolished for DCs + MVs_DG75_ (Figure 5B).

As expected, DCs directly loaded with the immunogenic peptide induced equal IFNγ T-cell-mediated production, independently by DPI treatment, confirming the specificity of the IFNγ response to the MUC1 peptide (Figure 5B).

These results show that the uptake of tumor MVs carrying endogenous source of radical species still sustains a mild MUC1 degradation favoring antigen translocation into the endoplasmic reticulum, albeit a fully competent DC is required so that optimal cross-presentation may occur.

## Discussion

Release of tumor-derived MVs is emerging as a crucial factor in mediating the complex cross talk among transforming cells, immune system and local microenvironment during tumor progression (7).

*In vivo*, tumor MVs most probably exert these distinct and apparently contradictory roles depending upon the stage of tumor transformation: initially, tumor-derived MVs deliver antigenic *repertoire* to DCs present in the tissue and resident in the adjacent lymph nodes contributing to *immunesurveillance* (26, 27). Then cell transformation and the immune sculpting processes give rise to transformed cell clones with high immune suppressive potential and tumor MVs shift their function mediating immunesuppression (28, 29).

The immunoactivatory role of MVs probably lies in their ability to reprogram DC functions, besides simply convey a tumor antigenic *repertoire* to APCs. DCs are activated by tumor MVs through delivery of tumor DNA that triggers a protective anti-tumor immune response *in vivo* (30, 31). Also indirect pieces of evidence suggest that MV internalization modulates antigen cross-processing ability of DCs. In fact, DCs appear more efficient in cross-priming antigens carried by MVs than the soluble antigens (11, 32) and only when carried by MVs, large tumor glycosylated antigens as MUC1 are cross-presented and CD8^+^ T cell responses are activated (16).

This biological mechanism may also pertain to other tumor-associated glycoproteins that compose for a large proportion the immunogenic *repertoire* of tumor cells.

These results prompted us to hypothesize that tumor MVs could act as signalosome for DCs, modifying the intracellular routing of the antigenic cargo and inducing HLA class I processing.

Dendritic cell phagosome appears to be a key cell compartment balancing the cross-processing of internalized exogenous antigens (3). Thus, we investigated the effect of MV internalization by DCs on phagosomal compartment, employing tumor-derived MVs obtained from ovarian cancer ascites fluid (MVs_Asc_) and from the MUC1-DG75 line (MVs_MUC1-DG75_).

A single cell simultaneously sheds several subsets of extracellular vesicles generated by distinct biogenesis processes and characterized by a specific size range and distinctive molecular cargo. Both MV samples contained different vesicle subsets heterogeneous for size as detected by NTA analysis. The tumor MVs had a mean size of 194 and 130 for MVs_Asc_ and MVs_MUC1-DG75_, respectively. This corresponded to the size of vesicles generated by membrane evagination (6). Biochemical characterization further suggested that vesicle samples were originated from distinct intracellular compartments, with a prevalence of vesicles derived from the plasma membrane/exocytic pathway. Both the tumor MV samples carried the MUC1 tumor-associated glycoprotein.

Following uptake of MVs_Asc_ by DCs, after 12 h, MUC1 was found in association with calreticulin and HLAI molecules, markers for HLA class I compartment, indicating that cross-processing occurred.

In addition, pulsing of DC cells with MUC1-carrying MVs induced a T-cell-mediated response evaluated as IFNγ production.

The cross-processing ability is an exquisite feature of DCs and it is determined by a finely tuned proteolytic activity: in fact, DCs exhibit a reduced capacity for phagosomal degradation than do other APCs. It is thought that the partial degradation of the internalized antigens occurring in the phagosomes sustains antigen stability. This promotes antigen export to the cytosol, where further degradation by proteasome occurs, generating the peptide *repertoire* for HLA class I loading (4). The alkalinization of phagosome environment regulates protease function and is finely tuned by a dynamic and balanced activity of NOX2 and the V-ATPase proton pump (24). *In vivo*, DCs with higher cross-presenting ability, such CD8α^+^ DCs, are characterized by phagosomes with alkaline microenvironment that is sustained by enhanced ROS accumulation (33). Conversely, inhibition of ROS production lowers phagosomal pH leading to block of cross-processing and presentation (34).

Indeed, transfer of the MUC1 antigen to DCs by MVs_Asc_ or MVs_MUC1-DG75_ induced a phagosomal ROS increase and a mild, but significant, phagosomal alkalinization in the first 30 min of chase.

The early alkalinization observed was dependent upon the ROS accumulation triggered by MV uptake. In fact, in DCs treated with the NOX2 inhibitor DPI with a consequent acid phagosomal pH, MV internalization restored phagosomal ROS and pH levels.

Internalization of the soluble rMUC1 did not induce such events as expected since MUC1 processing is confined to HLA class II compartment (16, 35). Also Liposomes internalization did not trigger ROS and pH increase, suggesting that the changes in the phagosomal environment were not a general response to vesicle uptake.

It has been shown that phagosomal ROS and pH increase occurs when soluble antigen is immobilized on inert material or onto polystyrene beads with a size >500 nm (36, 37). This does not apply to our system both for the composition and for the size of MVs and Liposomes employed (size <500 nm) suggesting that the molecular cargo of tumor MVs could be responsible for the modification induced at DC phagosomal level.

So far, biochemical and proteomic pieces of evidence indicate that radical species as well as redox enzymes can be found in diverse vesicle subsets from a variety of cell types (38, 39). The cargo radical activity of MVs has shown to be relevant in biological processes of diverse type of cells and tissues as such as endothelial barrier, cardiomyocytes, and epithelial cells (40–42).

Indeed both MVs_Asc_ and MVs_MUC1-DG75_ were source of radical species as tested by staining with DCF-DA, a probe sensitive to radicals (43). It is unlikely that the oxidant anions *per se* could make account for the phagosomal ROS increment observed in MVs pulsed DCs due to their high instability (i.e., the isolation and storage processes could dampen anion reactivity). It is conceivable that MVs might carry enzymes producing radical species. This hypothesis would be also in agreement with the kinetics of ROS triggering and pH early increase that we observed. Recently, it has been shown that NOX2 carried by MVs is functional in producing ROS and mediating Treg functions (44). Interestingly, preliminary data indicate that NOX2 was present in both MVs_Asc_ and MVs_MUC1-DG75_ (data not shown) and ongoing studies are currently being carried out to investigate the redox enzyme(s) that can be involved in this process.

Nevertheless, MVs molecular *repertoire* by itself does not appear to be sufficient to translate phagosomal alkalynization in optimal antigen presentation. Indeed, MVs internalization significantly restored ROS and pH levels in DPI-treated DCs (2 h). Prolonged DPI treatment of DCs (14 h) drastically decreased, but not abolished, MUC1 translocation to ER/HLAI. Concurrently, a dampened activation of MUC1 antigen-specific CD8^+^ T cell response was observed.

These results suggest that while MV internalization could be a triggering event, the recipient DCs need to be equipped with a competent antigen-processing machinery to activate an efficacious antigen presentation.

Preliminary results are indicative that phagosome maturation process may be differently modulated by tumor MV uptake, not by Liposomes (data not shown).

The phagosome is a central cellular hub for docking and sorting of antigens, fated to degradative/antigen presenting intracellular pathways. The cross talk among these distinct compartments is crucial for the antigen presentation machinery, although the mechanisms underlying cross-presentation are still unclear (45). Several molecules, key regulators in the protein import/export from intracellular compartments, seem to play a critical role in antigen cross-priming. Recently, Sec22b, a SNARE family member modulating the protein trafficking from ER to the endocytic and phagocytic pathways, has been shown to play a crucial role in antigen cross-presentation for effective anti-tumor immunity *in vivo* (46).

It is also clear that cross-priming is the final event of a metabolic and tightly regulated reprogramming of the DCs, also in response to extracellular *stimuli*.

In DCs, the mTOR network integrates extracellular molecular cues, such as nutrient, stress, PAMPs, and DAMPs, and triggers transcriptional and metabolic reprogramming of DCs (47). The balance between cross-presentation of exogenous antigens and lysosome activity in DCs is promptly switched by the transcription factor EB (TFEB), regulated by mTOR pathway (48).

It is interesting to note that mTOR, TFEB, and molecules involved in intracellular protein trafficking such as Sec22b are found in tumor-derived MVs (49–52).

Whether tumor MVs may activate other metabolic processes resulting in phagosomal alkalinization, as early response, and then cross-processing, as late event, remains to be determined and further investigations are required to decipher the complex role that tumor MVs play in the cross talk among tumor, immune cells, and tissue microenvironment.

At our knowledge, this is the first evidence that tumor MVs have been described to reprogram DC antigen-processing machinery by ROS-mediated mechanism, inducing early alkalinization of the phagosomal compartment and sustaining antigen cross-processing.

This mechanism can be relevant for shaping antigen immunogenicity *in vivo*, also for those large tumor glycosylated antigens as such as MUC1. These findings may further support the exploitation of tumor-derived MVs for the design of cell-free-based immunogens for anti-cancer therapy (53).

## Ethics Statement

This study was approved by the Ethical Committee of Sapienza University of Rome—Policlinico Umberto I University Hospital (Protocol number: 4214/2016) and carried out in accordance with its recommendation. Sampling of biological materials from cancer patients (ovarian ascites fluid or blood samples) was approved by the Ethical Committee (Protocol number: 1454/2008). Triple-peptide vaccination clinical protocol was approved by Ethical Committee and authorized by Italian National Institute of Health: protocol n. LITRM/DIMIGE05/01. Written informed consent was obtained from all the subjects in accordance with Declaration of Helsinki.

## Author Contributions

All authors contributed with their specific expertise to study design, data collection, analysis, and interpretation of results and to critically evaluate and approve the manuscript prior publication. AR and FeB designed the study and the experiments and wrote the manuscript. FeB and CN developed the methodology. PBP, IR, and FiB collected blood and ascites fluid samples and clinical information. HRK, IGZ, CN, and IR performed microvesicle production, isolation, and biochemical characterization. HRK performed CD8^+^ T cell culture and IFNγ-ELISPot. MRT and FrB were responsible for the immunofluorescence studies. GC and SP provided liposomes and their characterization. FA and MN provided valuable support for the study design and interpretation of results. AR supervised the study.

## Conflict of Interest Statement

The authors declare that the research was conducted in the absence of any commercial or financial relationships that could be construed as a potential conflict of interest.
