# Supplementary material for: Tumor-Derived Microvesicles Modulate Antigen Cross-Processing via Reactive Oxygen Species-Mediated Alkalinization of Phagosomal Compartment in Dendritic Cells
Source: Front Immunol. 2017 Sep 25;8:1179. doi: 10.3389/fimmu.2017.01179 (PMC5622295; doi:10.3389/fimmu.2017.01179)
Supplement: Supplementary file 1 [file Image_1.pdf]

Tumor-derived Microvesicles modulate antigen cross-processing via ROS-mediated alkalinization of phagosomal compartment in Dendritic Cells

Federico Battisti, Chiara Napoletano, Hassan Rahimi Koshkaki, Francesca Belleudi, Ilaria Grazia Zizzari, Ilary Ruscito, Sara Palchetti, Filippo Bellati, Pierluigi Benedetti Panici, Maria Rosaria Torrisi, Giulio Caracciolo, Fabio Altieri, Marianna Nuti, Aurelia Rughetti*

*Corresponding author: Aurelia Rughetti; Department of Experimental Medicine, "Sapienza" University of Rome, Viale Regina Elena, 324, 00161 Rome, Italy; phone: +39 0649973025; fax: +39 0649973029; email: aurelia.rughetti@uniroma1.it

A

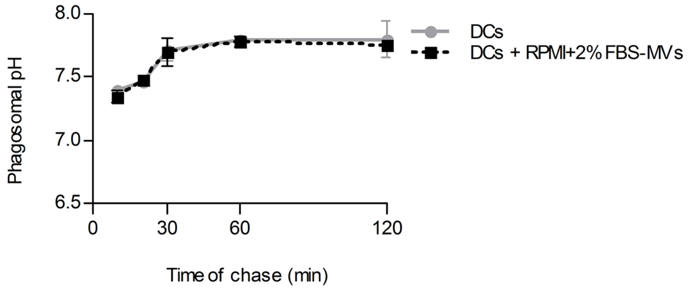

B

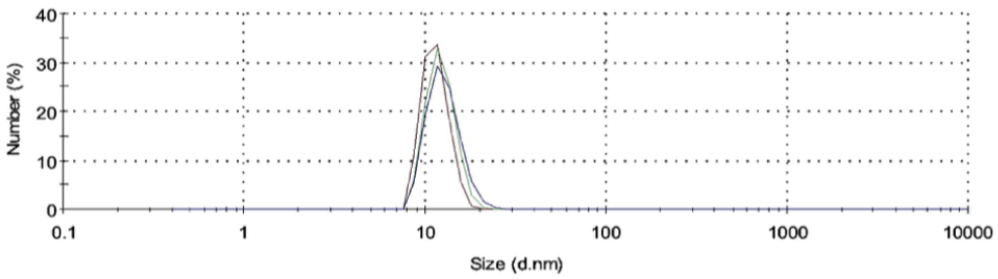

Supplementary Figure 1: DC phagosomal pH is not affected by serum bovine origin present in the culture medium that does not contain MVs

A) MVs purified from the cell culture media (deriving from the FBS) were employed to pulse DCs. DCs (grey line) and DCs + RPMI+2%FBS-MVs (black line) show similar phagosomal pH kinetics. The average and standard deviation (SD) of 3 different independent experiments are shown.

B) Size measurement of ultracentrifuged culture medium (RPMI+2%FBS) using Zetasizer Nano ZS90 spectrometer (Malvern Instruments Ltd). The three curves show three independent measurements for the pellet of ultracentrifuged RPMI+2%FBS. These size distributions were not compatible with the presence of vesicles, but they were the typical size distribution of proteins enriching culture media.
