# Supplementary material for: Tumor-Derived Microvesicles Modulate Antigen Cross-Processing via Reactive Oxygen Species-Mediated Alkalinization of Phagosomal Compartment in Dendritic Cells
Source: Front Immunol. 2017 Sep 25;8:1179. doi: 10.3389/fimmu.2017.01179 (PMC5622295; doi:10.3389/fimmu.2017.01179)
Supplement: Supplementary file 2 [file Image_2.pdf]

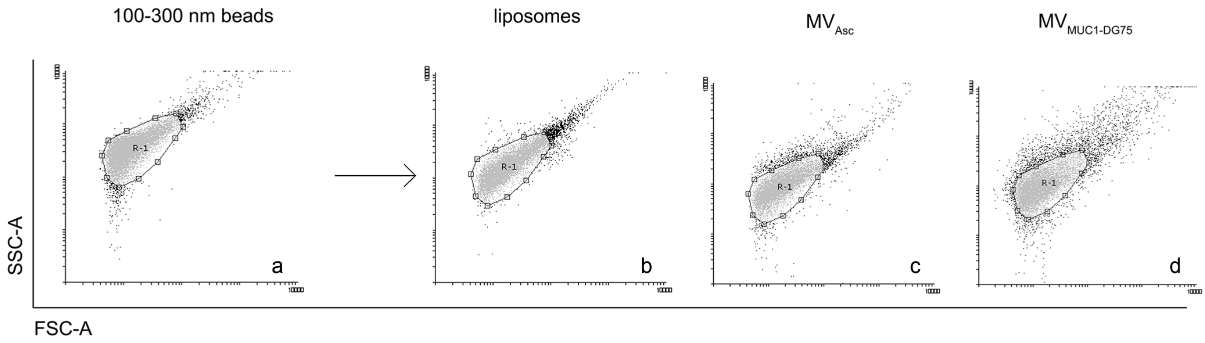

Supplementary Figure 2: Analysis of Liposomes and tumor derived MVs by flow cytometry

Fluorescent Nile Red particles particles with size between 100-300 nm were used as reference (panel a) for the gating of Liposomes and microvesicle samples. Gating of Liposomes (panel b), MVs_{Asc} (panel c) and $MVs_{MUC1\text{-}DG75}$ (panel d). Results are reported in dot plot. x axis: FSC-A; y axis: SSC-A.
