# Supplementary material for: Tumor-Derived Microvesicles Modulate Antigen Cross-Processing via Reactive Oxygen Species-Mediated Alkalinization of Phagosomal Compartment in Dendritic Cells
Source: Front Immunol. 2017 Sep 25;8:1179. doi: 10.3389/fimmu.2017.01179 (PMC5622295; doi:10.3389/fimmu.2017.01179)
Supplement: Supplementary file 3 [file Image_3.pdf]

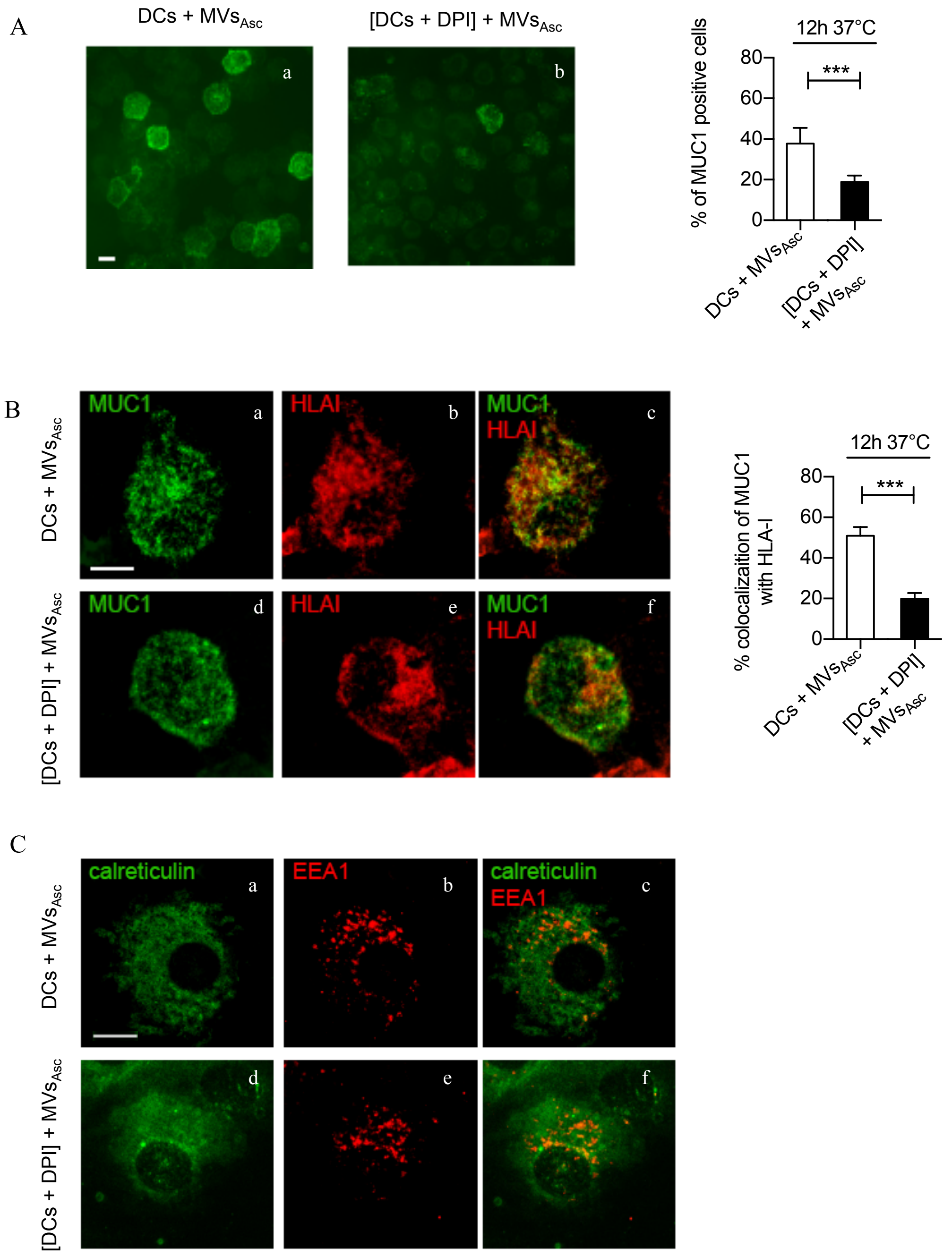

Supplementary Figure 3: DPI-treated DCs shows a modified MUC1 distribution

A) Intracellular localization of MUC1 carried by MVs_{Asc} in DCs (a) and in DPI-treated DCs (b) was visualized by immunofluorescence staining after 12 hours of internalization employing the anti-MUC1 MoAb Ma552. The percentage of $MUC1^{+}$ DCs was calculated analyzing 5 different experiments. Results are expressed as mean values ± SD. Student *t test* was performed and significance level has been defined as ***$p<0.005$ versus the corresponding DPI-untreated DCs.

B) Intracellular localization of MUC1 carried by MVs_{Asc} in DCs (a-c) and in DPI-treated DCs (d-f) was visualized by immunofluorescence staining after 12 hours of internalization employing the anti-MUC1 MoAb Ma552 (green, a, c, d and f) and the anti-HLAI MoAb W6/32 (culture supernatant; red, b, c, e and f). Colocalization of the immunofluorescence signals is shown (yellow, c and f). Quantitative analysis of colocalization was calculated as reported in materials and methods and plotted as histograms. Results are expressed as mean values ± SD. Student *t test* was performed and significance level has been defined as $p<0.05$. ***$p<0.005$. Bar: 10 µm.

C) Intracellular localization of Calreticulin and EEA1 in DCs (a-c) and in DPI-treated DCs (d-f) pulsed with MVs_{Asc} was visualized by immunofluorescence staining after 12 hours of internalization employing anti-calreticulin polyclonal antibody (green, a, c, d and f) and anti-EEA1 MoAb (red, b, c, e and f). No colocalization was observed. Bar: 10 µm.
